# Evolutionary effects of nitrogen are not easily predicted from ecological responses

**DOI:** 10.1002/ajb2.16095

**Published:** 2022-11-13

**Authors:** Joseph Waterton, Mark Hammond, Jennifer A. Lau

**Affiliations:** ^1^ Department of Biology Indiana University 1001 E. 3rd St. Bloomington IN 47405 USA; ^2^ Kellogg Biological Station Michigan State University Hickory Corners MI 49060 USA; ^3^ Department of Biology and the Environmental Resilience Institute Indiana University 1001 E. 3rd St. Bloomington IN 47405 USA

**Keywords:** herbivory, light asymmetry, LTER, nitrogen enrichment, opportunity for selection, phenotypic selection, Poaceae, *Setaria faberi*, species diversity

## Abstract

**Premise:**

Anthropogenic nitrogen (N) addition alters the abiotic and biotic environment, potentially leading to changes in patterns of natural selection (i.e., trait–fitness relationships) and the opportunity for selection (i.e., variance in relative fitness). Because N addition favors species with light acquisition strategies (e.g., tall species), we predicted that N would strengthen selection favoring those same traits. We also predicted that N could alter the opportunity for selection via its effects on mean fitness and/or competitive asymmetries.

**Methods:**

We quantified the strength of selection and the opportunity for selection in replicated populations of the annual grass *Setaria faberi* (giant foxtail) growing in a long‐term N addition experiment. We also correlated these population‐level parameters with community‐level metrics to identify the proximate causes of N‐mediated evolutionary effects.

**Results:**

N addition increased aboveground productivity, light asymmetry, and reduced species diversity. Contrary to expectations, N addition did not strengthen selection for trait values associated with higher light acquisition such as greater height and specific leaf area (SLA); rather, it strengthened selection favoring lower SLA. Light asymmetry and species diversity were associated with selection for height and SLA, suggesting a role for these factors in driving N‐mediated selection. The opportunity for selection was not influenced by N addition but was negatively associated with species diversity.

**Conclusions:**

Our results indicate that anthropogenic N enrichment can affect evolutionary processes, but that evolutionary changes in plant traits within populations are unlikely to parallel the shifts in plant traits observed at the community level.

Nitrogen (N) is the most limiting resource on land and a major driver of primary productivity and plant community composition (Elser et al., 2007; LeBauer and Treseder, 2008; Borer et al., 2014; Du et al., 2020). However, while community‐level effects of N enrichment have long been a major focus of research (reviewed by Cleland and Harpole, 2010), concurrent effects on evolutionary processes within populations are relatively understudied. Such evolutionary consequences will be mediated to a large extent by changes in (1) patterns of natural selection (i.e., trait–fitness relationships) and (2) the opportunity for selection (variance in relative fitness; I) that limits the maximum rate of adaptive evolution. As we detail below, N is expected to alter natural selection on populations for the same reason it affects plant communities—higher N is likely to favor different traits than are favored in lower N environments. Nitrogen also is expected to affect the opportunity for selection via its effects on mean fitness (many species benefit from N enrichment) and also variation in fitness.

Nitrogen has particularly strong effects on plant communities. As a result, it essentially changes the “ecological theater”, providing multiple potential avenues for influencing the “evolutionary play” acted out by plant populations (Figure 1). First, N enrichment increases primary productivity (Gough et al., 2000; Suding et al., 2005; Isbell et al., 2013; Avolio et al., 2014; Borer et al., 2014; Stevens et al., 2015). Second, this increase in productivity is associated with shifts in light availability, with N enrichment shifting communities from N limited to light limited. Third, chronic N enrichment consistently decreases plant species diversity and increases the dominance of non‐native plant species (Gough et al., 2000; Crawley et al., 2005; Suding et al., 2005; Isbell et al., 2013; Avolio et al., 2014; Seabloom et al., 2015; DeMalach et al., 2017b), with the abundance of certain species increasing (hereafter “enriched” species; La Pierre and Smith, 2015) at the expense of others that decrease in abundance or are extirpated (hereafter “baseline” species; La Pierre and Smith, 2015). This decline in diversity is likely due the increased light asymmetry observed in high N. The light asymmetry hypothesis (Newman, 1973) proposes that changes in community composition occur because soil N becomes less limiting to plant growth, increasing aboveground primary productivity and shifting the predominant mode of competition from size‐symmetric belowground competition for N to size‐asymmetric aboveground competition for light (meaning that taller individuals receive disproportionately more light per unit size; Weiner, 1990; Schwinning and Weiner, 1998; Hautier et al., 2009). The increasing predominance of size‐asymmetric light competition under N enrichment causes exclusion of shorter, slower‐growing baseline species (Hautier et al., 2009; DeMalach et al., 2016, 2017a, 2017b; Zhang et al., 2020; Xiao et al., 2021). Nitrogen enrichment also may reduce species diversity via other, nonmutually exclusive mechanisms, such as reducing the number of limiting resources and thus the dimensionality of niche space (niche dimension hypothesis; Harpole and Tilman, 2007). Finally, N enrichment can alter biotic interactions with higher trophic levels, as increases in productivity and/or tissue N content can increase herbivory intensity (Nams et al., 1996; reviewed by Stevens et al., 2018). These community‐level impacts of N enrichment thus potentially represent large shifts in environmental factors that are also likely selective agents shaping patterns of natural selection and the opportunity for selection.

**Figure 1:**
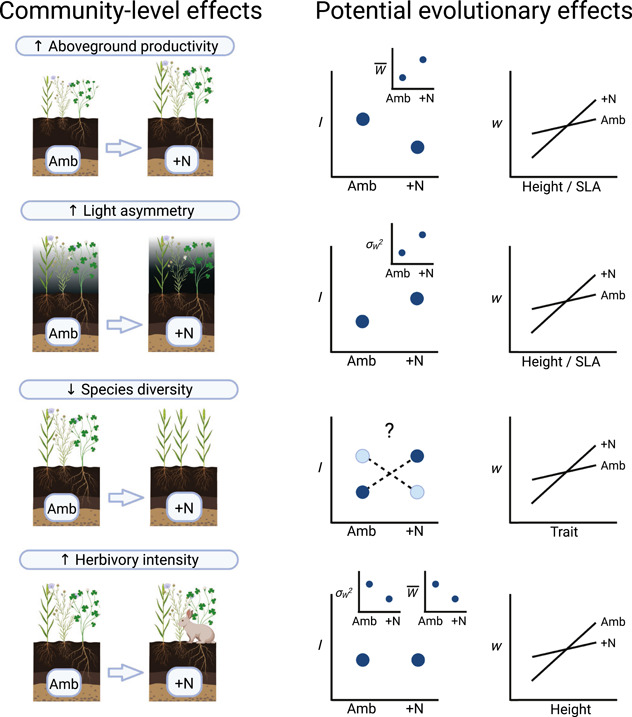
Conceptual diagram showing community‐level impacts of N addition (+N) and their potential effects on evolutionary processes relative to ambient conditions (Amb). N addition typically increases annual net primary productivity (ANPP) and mean fitness ($\bar{W}$) of “enriched” species like *Setaria faberi*, which theory predicts will reduce the opportunity for selection (I). We also predict increased selection on light acquisition traits with N addition (i.e., traits have larger effects on relative fitness [*w*]). N addition also increases light asymmetry because of increased ANPP. We predict that increased light asymmetry will increase the variance in fitness ($\sigma_{W}^{2}$) because taller plants will receive disproportionately more light, resulting in increased opportunity for selection. As with ANPP, we predict that increased light asymmetry will increase selection favoring light acquisition traits. N addition reduces species diversity, and this reduced diversity may either increase or decrease variance in fitness (and, therefore, the opportunity for selection), depending on whether more consistent competitive interactions increase or decrease growth disparities among conspecifics. Reduced diversity may also increase the strength of selection as the focal species experiences more consistent selection from one or a few competitors rather than diffuse selection from competitive interactions with many different species. Finally, N addition typically increases herbivory intensity, which typically reduces mean fitness but might also reduce the variance in fitness as taller, better performing plants may be preferred by herbivores. Because the opportunity for selection depends on both mean fitness and variance in fitness, any effects of N mediated by herbivory will depend on the relative magnitude of effects on these two properties. If herbivores prefer taller individuals, then selection for increased height might be reduced under N addition as herbivores reduce the fitness advantage of taller individuals. Created with BioRender.com.

The strength and direction of natural selection is determined by the relationships between traits and relative fitness (w; absolute fitness [W] divided by population mean fitness [$\bar{W}$]), and the environment determines those trait–fitness relationships (i.e., whether traits are favored or disfavored in a particular environment). Meta‐analysis shows that variation in the abiotic and biotic environment substantially alters selection on a wide range of plant traits (Caruso et al., 2020). However, studies investigating the effects of N on natural selection have largely focused on floral traits in animal‐pollinated species, finding that selection on floral morphology and phenology often differs with N addition (e.g., Caruso et al., 2005; Case and Ashman, 2007; Sletvold et al., 2017). Few studies have directly tested how N enrichment affects natural selection on other traits, such as those influencing resource acquisition. Yet, given that N enrichment often favors species with trait values associated with high light acquisition, such as greater height, leaf area, and specific leaf area (leaf area/dry mass, hereafter SLA; La Pierre and Smith, 2015; Siefert and Ritchie, 2016; Tatarko and Knops, 2018), it is plausible that N enrichment could favor those same traits within species (i.e., intensify selection on those traits; Figure 1). Moreover, variation in light availability, species diversity, and herbivory have all been shown to alter patterns of natural selection (McGoey and Stinchcombe, 2009; van Moorsel et al., 2018b; Waterton and Cleland, 2021), suggesting that N enrichment may be an important selective agent indirectly via its effects on these community‐level properties. Consistent with this suggestion, Petipas et al. (2020) found that N addition strengthened selection favoring lower root to shoot ratios in switchgrass (*Panicum virgatum* L.), likely reflecting a shift from N limitation to light limitation with N addition. Many additional aboveground functional traits, including morphological (e.g., height, leaf count, SLA) and phenological traits (e.g., flowering date), influence resource acquisition and plant performance in response to variation in soil nutrients (e.g., Ryser and Lambers, 1995; Nord and Lynch, 2008; Wang et al., 2016), yet measurements of how N affects selection on these traits are scarce.

The variance in relative fitness, known as the opportunity for selection (I; Crow, 1958) influences a population's evolutionary potential because it limits both the maximum strength of selection that can act on any given trait and thus the rate at which fitness can increase over time. The opportunity for selection sets the upper boundary for the strength of selection, although selection rarely reaches this limit since traits and relative fitness are rarely perfectly correlated (Shuster and Wade, 2003). However, the opportunity for selection remains useful because it provides information on how selection can act even on unmeasured traits (Krakauer et al., 2011). In addition, differences in the strength of selection between environments can result from differences in either (1) trait–fitness correlations or (2) the opportunity for selection; therefore, quantifying the opportunity for selection is useful for understanding the drivers of variation in selection.

Because relative fitness equals absolute fitness divided by population mean fitness, the opportunity for selection equals the ratio of variance in absolute fitness to squared mean absolute fitness (Wade and Shuster, 2005), and thus, any change in this ratio will change the opportunity for selection. Environmental variation strongly influences the opportunity for selection; for example, environmental stresses such as low light increased the opportunity for selection in *Sinapis arvensis* (Stanton et al., 2000). High‐resource environments, such as those enriched in N, have been predicted to decrease the opportunity for selection on the basis that they often increase mean fitness (Rundle and Vamosi, 1996). This prediction assumes that gains in absolute fitness are spread sufficiently evenly among individuals in the population in absolute terms (e.g., all individuals produce 10 more offspring). Yet, increases in mean fitness could increase, decrease, or have no effect on the opportunity for selection depending on changes in fitness variance (further discussion and hypothetical examples are given in Appendix S1; Reiss, 2012; Fugère and Hendry, 2018). Nitrogen enrichment can also affect the opportunity for selection because the increased predominance of size‐asymmetric light competition could exacerbate growth differences to increase absolute fitness variance (Weiner, 1990; Figure 1). In contrast, the increased grazing intensity commonly observed in N‐enriched communities could disproportionately impact larger, more apparent individuals to decrease absolute fitness variance (Waterton and Cleland, 2021; Figure 1).

Combining experimental manipulations of environmental factors, such as N, in the field with individual‐level measurements of traits and fitness is a powerful approach for testing hypotheses about how environmental factors alter evolutionary trajectories (Wade and Kalisz, 1990). Although evolutionary responses to variation in soil N can be studied by examining clinal trait variation along natural environmental gradients (e.g., Kichenin et al., 2013; Brouillette et al., 2014), such studies cannot identify causal selective agents responsible for trait differences among populations because variation in N may be confounded with other environmental variables. Experimentally manipulating N avoids this issue of causality (Wade and Kalisz, 1990), and doing so under field conditions is ideal because more controlled conditions will likely increase bias in results due to differences in, or the absence of, selective agents, such as drought stress (cf. Warwell and Shaw, 2018), pathogens (cf. Brunet and Mundt, 2000), and herbivores (cf. Waterton and Cleland, 2021). Experiments in long‐term ecological research (LTER) sites, in which N has been applied continuously for many years (e.g., decades), present a unique opportunity to characterize how N enrichment shapes plant evolution because N‐induced ecological effects that might mediate evolutionary effects (e.g., changes in species diversity) can take years to manifest (reviewed by Smith et al., 2009).

In this study, we investigated the evolutionary consequences of N enrichment in the non‐native annual grass *Setaria faberi* Herrm. (giant foxtail, Poaceae), a species that has increased its annual productivity by an average of 144% in response to long‐term N addition (~30 years) in the Kellogg Biological Station LTER site (Hickory Corners, Michigan, USA; https://lter.kbs.msu.edu/datatables/154). We tested the hypothesis that N enrichment influences patterns of natural selection and the opportunity for selection via effects on mean fitness, species diversity, light asymmetry, and herbivory intensity. Specifically, we predicted that (1) N addition would strengthen selection for trait values associated with greater light acquisition and that (2) N addition influences the opportunity for selection by increasing both mean fitness and the variance in absolute fitness, with the latter resulting from stronger size‐asymmetric light competition. We measured functional traits and fitness in naturally occurring *S. faberi* in plots with ambient conditions or long‐term N addition and in which we quantified community metrics that might potentially mediate N effects on evolution: primary productivity, light asymmetry, species diversity, and herbivory intensity.

## MATERIALS AND METHODS

### Study species

*Setaria faberi* is an annual grass native to eastern Asia (Wang et al., 1995) that has spread across the globe due to human activity. This species has become naturalized in eastern North America following its introduction as a contaminant in imported grains in the 1920s (Nurse et al., 2009). *Setaria faberi* is an early‐successional species that can be found in a wide range of habitats including crops, roadsides, gardens, and old fields (Nurse et al., 2009). Plants can reach 2.5 m in height, with either single stems or multiple stems branching from the base (Nurse et al., 2009). Each plant typically produces one long spike‐like panicle at the top of each stem but can sometimes produce additional smaller panicles along the stem (M. Hammond, personal observation). *Setaria faberi* is largely self‐fertilizing, with outcrossing rates between 0 and 2.37% (Volenberg and Stoltenberg, 2002). As an annual, total fruit (technically, caryopsis) production per individual (hereafter “fecundity”, see below), represents a measure of lifetime fitness via the female function.

### Study site

We carried out the study in the Kellogg Biological Station LTER site in Hickory Corners, Michigan, USA (42.41°N, 85.37°W) from June to October 2020. We studied naturally occurring *S. faberi* individuals in the annually tilled 20 × 30 m portion of the six replicate 1‐ha early‐successional plots that have been maintained since 1989. Plots are tilled annually in the spring (typically in late March to early May) to maintain early successional communities and are dominated by annual herbaceous species including *S. faberi*, *Ambrosia artemisiifolia* L. (common ragweed), *Cyperus esculentus* L. (yellow nutsedge), and *Digitaria sanguinalis* (L.) Scop. (hairy crabgrass). The tilled portion of each plot contains two 5 × 5 m subplots, spaced 5 m apart, each subplot belonging to one of two N treatments: “addition” and “ambient”. Addition subplots receive 123 kg N ha^−1^ y^−1^, supplied in a single application of either ammonium nitrate or urea fertilizer after germination of the annual community and immediately before a rain event (typically in late May to early July), while ambient subplots receive no fertilizer. In 2020, primary tillage with a chisel plow occurred on 5 May, secondary tillage occurred on 7 May, and urea was applied on 9 July.

### Field experiment

On 15 June, we marked approximately 100 putative *S. faberi* seedlings within a 0.5‐m wide strip in the northern side of each ambient and addition subplot (*N* = 1125 seedlings; final *N* = 974 due to misidentification of some seedlings [13.4%]), with a minimum 0.5‐m buffer between focal individuals and subplot edges. To capture a representative sample of natural phenotypic variation, we selected focal plants in each of four seedling height categories: (1) >10 cm; (2) >5–10 cm; (3) 2–5 cm; (4) <2 cm. Although we attempted to mark approximately equal numbers of individuals in each seedling height category, the smallest category in particular was underrepresented due to lower abundance (>10 cm: 24.9%; >5–10 cm: 34.8%; 2–5 cm: 33.6%; <2 cm: 6.8%).

We measured four traits: height, leaf count, SLA, and date of first flowering. From 16–25 July, we measured the height of the extended plant from the ground to the tip of the highest leaf, and we counted all visible leaves regardless of color or damage extent. We quantified SLA between 14–19 September, by collecting the youngest undamaged leaf and placing it in a sealed plastic bag with moist paper towels until scanning with a LI‐3000A portable area meter (LI‐COR, Lincoln, NE, USA) to obtain the surface area to the nearest square millimeter. We then dried leaves at 50°C for at least 72 h before weighing them to the nearest 0.01 mg and calculated SLA as leaf area (mm^2^) divided by leaf mass (mg). We also scored leaves for color to confirm that leaf senescence was not driving variation in SLA (full details are given in Appendix S2). We monitored each plant for flowering every 5–6 days between mid‐July until the end of October and used the date on which anthers were first visible as the date of first flowering. We estimated flowering dates intermediate between surveys based on the developmental stage of flowers.

We used total fruit count as a measure of lifetime fitness (via female function) for each individual. We collected mature fruits from each individual every 5–6 days between 6 September and 22 October by gently hand‐brushing panicles to dislodge mature fruits, repeating this process until all fruits had been collected from each plant. For plants that produced additional smaller panicles along the stem (*n* = 47, 4.8% of plants), we collected these fruits separately from those from the main stem‐tip panicle but included them in the total fruit count. We excluded 29 individuals from fitness analyses due to handling errors but included these individuals in trait‐only analyses. Because some fruits may have fallen between collections on individual plants, we also measured the total length of main (i.e., stem‐tip) panicles for each individual between 5–9 October. Main panicle fruit count correlated with main panicle length (Pearson correlation coefficient: *r* = 0.81, *P* < 0.001, *n* = 714; Appendix S3) indicating that our fruit collection procedure likely produced robust estimates of fitness.

We quantified four community metrics in each subplot: (1) total annual net primary productivity (ANPP), (2) light asymmetry (slope [β] of a linear regression of log photosynthetically active radiation [PAR, μmol m^−2^ s^−1^] against height [cm]), (3) species diversity (Shannon–Wiener diversity index; $H^{\prime }$) based on species‐level ANPP, (4) herbivory intensity (percentage of focal plants with inflorescences consumed). Full details of community metric data collection are given in Appendix S2. Briefly, we harvested aboveground biomass between 22 and 29 September (approximately at peak biomass) from 0.5 × 2 m strips within each subplot, sorted to species, and weighed after drying to calculate total ANPP and $H^{\prime }$. We measured PAR using an AccuPAR LP80 PAR Ceptometer (Decagon Devices, Pullman, WA, USA) at 5 heights: ground level, 25%, 50%, and 75% canopy height, and at the canopy top within an hour of the solar zenith on clear days between 30 August and 4 September. We recorded the presence/absence of mammalian herbivory on focal plant inflorescences and stems (e.g., entirely or partially clipped) throughout the growing season. Total ANPP was strongly correlated with light asymmetry (*r* = 0.89, *P* < 0.001); therefore, we only tested the effects of light asymmetry, species diversity, and herbivory intensity on patterns of selection and the opportunity for selection.

### Statistical analyses

We conducted all statistical analyses using R version 4.1.0 (R Core Team, 2021). We fit generalized linear mixed models (GLMMs) using the glmmTMB function in the package glmmTMB (Brooks et al., 2017) and linear mixed models (LMMs) using the lmer function in the package lme4 (Bates et al., 2015). To determine the suitability of models and data transformations, we used the simulateResiduals function in the package DHARMa (Hartig and Lohse, 2019). We evaluated the significance of fixed effects in mixed models with Type III Wald *χ* ^2^ tests using the Anova function in the package car (Fox and Weisberg, 2011). We report estimated marginal means (EMMs) in N treatments for means based on unbalanced individual‐level data within subplots, which we obtained using the emmeans package (Lenth et al., 2022). We made figures using the packages ggplot2 (Wickham, 2016) and patchwork (Pederson, 2020).

#### Effects of N on community metrics

We tested the effects of N addition on each community metric with separate LMMs or GLMMs. For total ANPP, species diversity, and light asymmetry, we fit LMMs in which subplot‐level values were predicted by N treatment with random intercepts for plot. We tested whether N addition influenced herbivory intensity with a binomial GLMM in which herbivory presence/absence on individual plants was predicted by N treatment, with random intercepts for plot and subplot. We used Bonferroni‐adjusted significance thresholds (*α* = 0.05/4) for these models to account for multiple comparisons.

#### Effects of N on mean trait values and fitness

We tested how N addition influenced mean trait values with LMMs. In each model, individual trait values (height, leaf count, SLA, flowering date) were predicted by N treatment with random intercepts for plot and subplot. To improve the normality of residuals, we square‐root‐transformed height and leaf count and log‐transformed SLA and flowering date. We used Bonferroni‐adjusted significance thresholds (*α* = 0.05/4) for these models to account for multiple comparisons.

Fecundity was zero‐inflated and overdispersed (Appendix S4); therefore, we used hurdle GLMMs to test how N treatment influenced mean absolute fitness for two separate fitness components: (1) survival to fruit production (“survival”); (2) total fruit count for surviving individuals (“fecundity”; Wadgymar et al., 2015; Waterton and Cleland, 2021). We chose to analyze fitness components with hurdle GLMMs because fecundity values would have otherwise violated the assumptions of standard linear modeling approaches (Mitchell‐Olds and Shaw, 1987; Schielzeth et al., 2020). We analyzed survival using binomial GLMMs with a logit link (zero part) and non‐zero values of fecundity with zero‐truncated negative binomial GLMMs with a log link (non‐zero part). To test the effect of N treatment on the mean values of survival and fecundity, we fit zero and non‐zero hurdle GLMM parts with the relevant fitness component predicted by N treatment and random intercepts for plot and subplot.

#### Effects of N and community metrics on phenotypic selection

We tested the effect of N treatment on patterns of linear and nonlinear total and direct selection for the four focal traits (height, leaf count, SLA, flowering date). Nonlinear selection was largely weak and nonsignificant, and rarely differed between N treatments; therefore, we report the methods and results for linear selection analyses in the main text, and we report the methods for nonlinear selection analyses in Appendix S2 and the corresponding results in Appendix S5. For all selection analyses, we standardized trait values to a mean of zero and standard deviation of one within subplots because this corresponds to the scale at which interactions would take place between individuals (De Lisle and Svensson, 2017). We relativized fecundity to a mean of one within subplots (fecundity divided by mean subplot fitness), including all individuals irrespective of survival. Total selection acting on a given trait results from both direct selection acting on that trait and selection acting on other correlated traits (i.e., indirect selection). We estimated standardized selection differentials (linear: *S*_*i*_) that describe total selection in each N treatment as the regression coefficients from separate LMMs of relative fecundity predicted by each trait, with random slopes for subplot. We estimated standardized selection gradients (linear: *β*_*i*_) that describe direct selection as the partial regression coefficients from a LMM of relative fecundity predicted by all four traits, with random slopes for subplot.

Due to violations of LMM assumptions that would otherwise render significance tests unreliable (Mitchell‐Olds and Shaw, 1987; Schielzeth et al., 2020), we obtained 95% confidence intervals for selection coefficients and the difference in selection coefficients between N treatments with nonparametric bootstrapping. For each N treatment, we used the nonparametric cases bootstrap to resample individuals within each subplot to generate 1000 bootstrap samples that maintain the clustered structure of the data using the cases_bootstrap function in the package lmeresampler (Loy et al., 2022). We used the percentile method to estimate 95% confidence intervals for selection coefficients as bootstrap distributions were approximately symmetric about their means (Efron and Tibshirani, 1993), with a selection coefficient deemed significant if the 95% confidence interval did not contain 0. To obtain 95% confidence intervals for the difference in selection coefficients between N treatments, we calculated the difference between the selection coefficients from each of the 1000 independent bootstrap samples in each N treatment. The percentile method was used to calculate the 95% confidence interval for the difference in selection coefficients, which were deemed to differ significantly between N treatments if the 95% confidence interval did not contain 0.

To test whether total and direct linear selection in each subplot was associated with community metrics (light asymmetry, species diversity, and herbivory intensity), we extracted standardized selection differentials (*S*_*i*_) and gradients (*β*_*i*_) from each subplot and used these as the response variable in three separate PERMANOVAs (one for each community metric) for both differentials and gradients (i.e., six total; full details are given in Appendix S2). We used Bonferroni‐adjusted significance thresholds (*α* = 0.05/3) for PERMANOVAs to account for multiple comparisons. In the case that PERMANOVA revealed significant effects of community metrics (see Results), we then fit separate univariate LMMs with selection differentials/gradients for each trait in subplots predicted by the relevant community metric and with random intercepts for plot.

#### Effects of N and community metrics on the opportunity for selection

We calculated the opportunity for selection in each subplot separately as the variance in within‐subplot relativized fitness (note that this is equivalent to dividing the variance in absolute fitness by the square of mean absolute fitness for each subplot). To test whether N enrichment influenced the opportunity for selection, we fit a LMM with the opportunity for selection predicted by N treatment and with random intercepts for plot. To investigate how each community metric influenced the opportunity for selection, we fit three LMMs with the opportunity for selection predicted by either subplot light asymmetry, species diversity, or herbivory intensity, with random intercepts for plot. We used Bonferroni‐adjusted significance thresholds (*α* = 0.05/3) for these models to account for multiple comparisons.

To test the extent to which the strength of linear selection was determined by the opportunity for selection, we used the absolute value of linear selection differentials and selection gradients in subplots as the response variable in two separate PERMANOVAs (one for each type of selection coefficient). In the case that PERMANOVA revealed significant effects of the opportunity for selection (see Results), we then fit separate univariate LMMs with selection differentials/gradients for each trait in subplots predicted by the opportunity for selection and with random intercepts for plot.

#### Effects of N on phenotypic correlations

To describe the multivariate phenotypes available to selection, we estimated phenotypic correlations in each N treatment while including incomplete observations (i.e., any individual with values for two or more traits). To test how N altered phenotypic correlations, we used phenotypic correlations in each subplot as the response variable in a PERMANOVA fit with the adonis2 function in the package vegan (Oksanen et al., 2020) with plot included as a blocking factor (full details are given in Appendix S2). In the case that PERMANOVA revealed significant effects of N treatment (see Results), we then fit separate univariate LMMs with phenotypic correlations for each trait‐pair in subplots predicted by N and with random intercepts for plot.

## RESULTS

### Effects of N on community metrics

**Figure 2:**
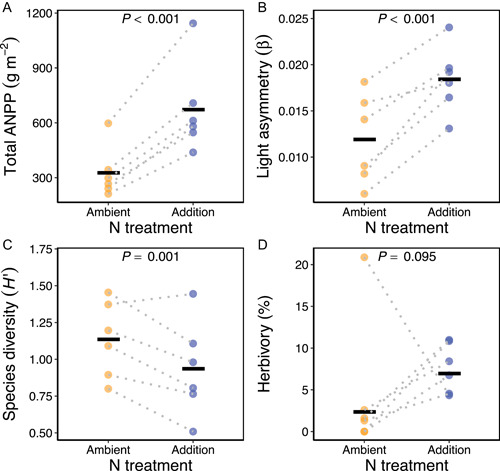
Mean values of (A) total annual net primary productivity (ANPP), (B) light asymmetry, (C) species diversity, and (D) herbivory intensity in ambient and addition N treatments. *P*‐values are from LMMs or GLMMs of each community metric predicted by N treatment. Solid horizontal lines show the means or estimated marginal means (herbivory only) across subplots. Filled circles show subplot values and dotted lines connect subplots in the same plot.

Nitrogen addition more than doubled ANPP ($\chi_{1}^{2}$ = 59.3, *P* < 0.001; Figure 2A), increased light asymmetry ($\chi_{1}^{2}$ = 30.7, *P* < 0.001; Figure 2B), and reduced species diversity ($\chi_{1}^{2}$ = 10.3, *P* = 0.001; Figure 2C). Herbivory intensity was 39% higher in the addition treatment, but this trend was nonsignificant ($\chi_{1}^{2}$ = 2.79, *P* = 0.095; Figure 2D).

### Effects of N on mean trait values and fitness

Nitrogen addition increased plant height by 52% (ambient EMM = 262 ± 31.0 mm, addition EMM = 397 ± 37.9 mm, $\chi_{1}^{2}$ = 28.0, *P* < 0.001; Appendix S6A), leaf count by 14% (ambient EMM =  5.32 ± 0.48, addition EMM = 6.05 ± 0.51, $\chi_{1}^{2}$ = 8.84, *P* = 0.003; Appendix S6B), and SLA by 11% (ambient EMM = 28.0 ± 1.07 mm^2^ mg^−1^, addition EMM = 31.1 ± 1.19 mm^2^ mg^−1^, $\chi_{1}^{2}$ = 9.58, *P* = 0.002; Appendix S6C), and delayed flowering by 4 days ($\chi_{1}^{2}$ = 27.0, *P* < 0.001; Appendix S6D). Nitrogen addition did not significantly affect the probability of surviving to produce fruits (ambient EMM = 84.3 ± 3.17%, addition EMM = 81.5 ± 3.43%, $\chi_{1}^{2}$ = 0.41, *P* = 0.52; Appendix S6E), but significantly increased mean fecundity by 267% for individuals that survived (ambient EMM = 28.9 ± 5.64, addition EMM = 106 ± 20.4, $\chi_{1}^{2}$ = 150.0, *P* < 0.001; Appendix S6F).

### Effects of N and community metrics on phenotypic selection

Estimates of direct and total selection in N treatments and the differences between them with 95% confidence intervals are provided in Table 1, but we summarize results here (values with asterisks are significantly different from 0 as assessed with 95% confidence intervals). Total selection consistently favored greater height (*S*_*i*_ ambient = 1.00*; *S*_*i*_ addition = 0.97*) and leaf count (*S*_*i*_ ambient = 0.93*; *S*_*i*_ addition = 0.83*), and earlier flowering (*S*_*i*_ ambient = –0.62*; *S*_*i*_ addition = –0.63*) across N treatments. Total selection favored lower SLA in both N treatments but was more than twice as strong in the addition treatment (*S*_*i*_ ambient = –0.42*; *S*_*i*_ addition = –0.86*; *S*_*i*_ difference = –0.45*). Direct selection also consistently favored greater height (*β*_*i*_ ambient = 0.93*; *β*_*i*_ addition = 0.97*) and leaf count (*β*_*i*_ ambient = 0.41*; *β*_*i*_ addition = 0.28*). Similar to the effect on total selection, N addition also strengthened direct selection favoring lower SLA with significant selection detected only in the addition treatment (*β*_*i*_ ambient = –0.050; *β*_*i*_ addition = –0.23*; *β*_*i*_ difference= –0.18*). In contrast to total selection, which consistently favored earlier flowering, direct selection favored later flowering in both N treatments (*β*_*i*_ ambient = 0.21*; *β*_*i*_ addition = 0.29*). With the exception of height, selection gradients were weaker or of the opposite sign to selection differentials (in the case of flowering time), indicating that correlations between traits contributed substantially to patterns of total selection (Table 1).

**Table 1:** Estimates of total and direct phenotypic linear selection coefficients and their differences for ambient and addition N treatments. Values are coefficients from models predicting within‐subplot relative fecundity (i.e., selection differentials [*S*_*i*_] for total selection and selection gradients [*β*_*i*_] for direct selection). We obtained 95% confidence intervals for selection coefficients and differences between N treatments with nonparametric bootstrapping. Values in bold indicate significant selection or selection that differed significantly between N treatments (i.e., 95% confidence interval does not contain 0).

Trait	N treatment		N‐mediated difference in selection coefficient (Addition – Ambient)
	Ambient	Addition	
**Total selection**			
Height (*S*_*i*_)	**1.00** (0.87, 1.13)	**0.97** (0.85, 1.08)	−0.028 (−0.21, 0.15)
Leaf count (*S*_*i*_)	**0.93** (0.78, 1.09)	**0.83** (0.70, 0.96)	−0.099 (−0.30, 0.11)
SLA (*S*_*i*_)	−**0.42** (−0.58, –0.28)	−**0.86** (−1.05, −0.72)	−**0.45** (−0.67, −0.23)
Flowering date (*S*_*i*_)	−**0.62** (−0.78, ‐0.52)	−**0.63** (−0.75, −0.53)	−0.008 (−0.17, 0.19)
**Direct selection**			
Height (*β*_*i*_)	**0.93** (0.70, 1.14)	**0.97** (0.76, 1.18)	0.042 (−0.27, 0.36)
Leaf count (*β*_*i*_)	**0.41** (0.18, 0.63)	**0.28** (0.085, 0.41)	−0.13 (−0.43, 0.12)
SLA (*β*_*i*_)	−0.050 (−0.20, 0.055)	−**0.23** (−0.43, −0.10)	−**0.18** (−0.39, −0.005)
Flowering date (*β*_*i*_)	**0.21** (0.050, 0.35)	**0.29** (0.15, 0.43)	0.080 (−0.10, 0.29)

**Figure 3:**
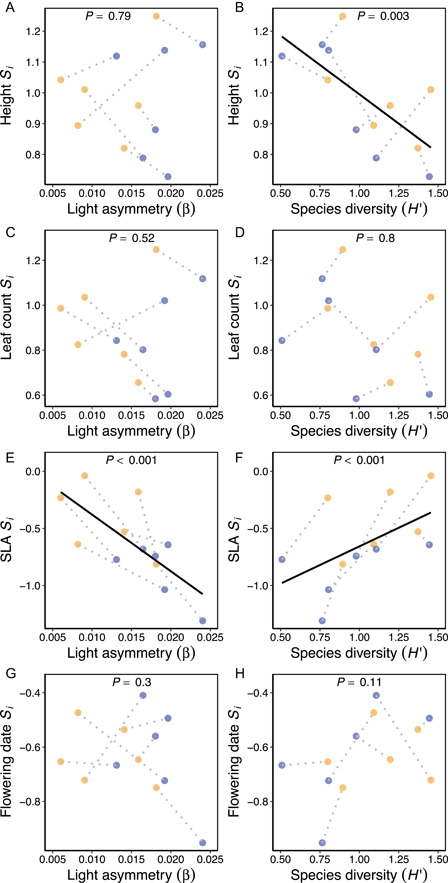
Associations between standardized selection differentials (*S*_*i*_) and light asymmetry and species diversity for (A, B) height, (C, D) leaf count, (E, F) specific leaf area (SLA), and (G, H) flowering date. *P*‐values are from separate LMMs of selection differentials predicted by light asymmetry or species diversity. Filled yellow and blue circles show subplot values in ambient and addition N treatments, respectively, and dotted lines connect subplots in the same plot. Solid lines indicate significant regressions.

Light asymmetry (pseudo‐*F*_1,10_ = 4.81, *P* = 0.016) and species diversity (pseudo‐*F*_1,10_ = 3.84, *P* = 0.016) affected the strength of selection differentials (*S*_*i*_). For light asymmetry, this overall effect was driven by increased light asymmetry increasing the strength of selection favoring lower SLA ($\chi_{1}^{2}$ = 16.7, *P* < 0.001, Figure 3E). For species diversity, this resulted because increased species diversity reduced selection on height ($\chi_{1}^{2}$ = 9.12, *P* = 0.003, Figure 3B) and SLA ($\chi_{1}^{2}$ = 29.5, *P* < 0.001, Figure 3F). Selection differentials did not vary with herbivory intensity (pseudo‐*F*_1,10_ = 1.19, *P* = 0.23). Standardized selection gradients (*β*_*i*_) for each subplot did not vary with light asymmetry (pseudo‐*F*_1,10_ = 1.16, *P* = 0.28), species diversity (pseudo‐*F*_1,10_ = 0.35, *P* = 0.84), or herbivory intensity (pseudo‐*F*_1,10_ = 0.80, *P* = 0.36).

### Effects of N and community metrics on the opportunity for selection

**Figure 4:**
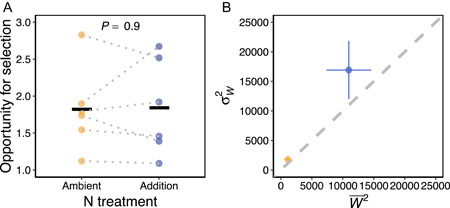
Effects of N addition on the opportunity for selection, visualized as (A) mean values across subplots in each treatment and (B) variance in absolute fitness ($\sigma_{W}^{2}$) against squared mean absolute fitness (${\overset{-}{W}}^{2}$) averaged across subplots in each treatment. (A) The *P*‐value is from a LMM of the opportunity for selection predicted by N treatment. Solid horizontal lines show the means across subplots. Filled circles show subplot values and dotted lines connect subplots in the same plot. (B) Filled yellow and blue circles show the means ± SE of squared mean absolute fitness (${\overset{-}{W}}^{2}$) and variance in absolute fitness ($\sigma_{W}^{2}$) across subplots in ambient and addition N treatments, respectively. The dashed line is the identity line at which the opportunity for selection = 1.

**Figure 5:**
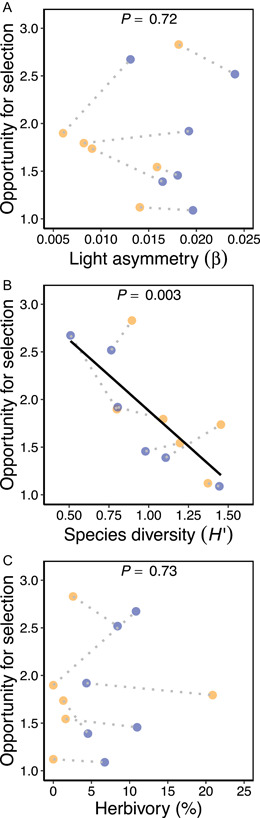
Effects of (A) light asymmetry, (B) species diversity, and (C) herbivory intensity on the opportunity for selection. *P*‐values are from LMMs of the opportunity for selection predicted by each community metric. Filled yellow and blue circles show subplot values in ambient and addition N treatments, respectively, and dotted lines connect subplots in the same plot. Solid lines indicate significant regressions.

Contrary to our expectations, the opportunity for selection was not significantly affected by N addition despite increases in mean fitness described above ($\chi_{1}^{2}$ = 0.015, *P* = 0.90; Figure 4). The opportunity for selection varied considerably among subplots within the same treatment, ranging between 1.12 and 2.83 in the ambient treatment and between 1.09 and 2.67 in the addition treatment, with plot explaining 77% of the variance after accounting for effects of N. The opportunity for selection was not predicted by light asymmetry ($\chi_{1}^{2}$ = 0.13, *P* = 0.72; Figure 5A) or herbivory intensity ($\chi_{1}^{2}$ = 0.12, *P* = 0.73; Figure 5C), but was significantly negatively associated with species diversity ($\chi_{1}^{2}$ = 8.70, *P* = 0.003; Figure 5B).

Across focal traits, the opportunity for selection did not significantly predict the strength of linear selection differentials (pseudo‐*F*_1,10_ = 5.72, *P* = 0.36) or gradients (pseudo‐*F*_1,10_ = 0.75, *P* = 0.66). This finding, combined with the lack of difference in the opportunity for selection between N treatments, suggests that the observed differences in the strength of natural selection were not due to differing limits to selection between N treatments.

### Effects of N on phenotypic correlations

Correlations among traits were strong, and N addition significantly strengthened phenotypic correlations among the four focal traits (pseudo‐*F*_1,10_ = 7.43, *P* = 0.031; Table 2). Univariate analyses of specific trait correlations showed that N increased the strength of correlations between SLA and height ($\chi_{1}^{2}$ = 8.69, *P* = 0.003; Table 2), leaf count ($\chi_{1}^{2}$ = 5.16, *P* = 0.023; Table 2), and flowering date ($\chi_{1}^{2}$ = 11.0, *P* < 0.001; Table 2). Phenotypic correlations and their statistical significance for each subplot are given in Appendix S7.

**Table 2:** Phenotypic correlations and their statistical significance across all individuals in ambient (above diagonal) and addition (below diagonal) N treatments. Values in bold indicate that phenotypic correlations differed significantly between N treatments. Phenotypic correlations and their statistical significance for each subplot are given in Appendix S7. Significance: ^†^ *P* < 0.1, *P < 0.05, **P < 0.01, ***P < 0.001.

Trait	Height	Leaf count	SLA	Flowering date
Height		0.46***	–0.19***	–0.56***
Leaf count	0.43***		–0.43***	–0.39***
SLA	–0.53***	–0.56***		0.27***
Flowering date	–0.63***	–0.43***	0.58***	

*Note*: SLA, specific leaf area.

## DISCUSSION

Anthropogenic N enrichment has wide‐ranging effects on plant community structure and ecosystem function (reviewed by Cleland and Harpole, 2010; Stevens et al., 2018), and it is plausible that the evolutionary consequences of N enrichment for constituent populations are equally pervasive. Documented clinal trait variation along natural N gradients suggests a potential role of this nutrient in driving selection (e.g., Kichenin et al., 2013; Brouillette et al., 2014), but isolating the evolutionary effects of N requires experimentation (Wade and Kalisz, 1990). We hypothesized that N addition alters patterns of natural selection and the opportunity for selection, in part because N addition favors suites of species with particular traits (e.g., traits associated with light acquisition) and causes shifts in putative selective agents such as species diversity, light asymmetry, and herbivory intensity. We found evidence that N altered selection in the annual *S. faberi* and that light‐asymmetry and diversity were associated with the strength of natural selection. Unexpectedly, N addition did not strengthen selection favoring trait values associated with greater light acquisition. We also found that, in contrast to theoretical predictions that increasing mean fitness lowers the opportunity for selection, N addition did not influence the opportunity for selection despite increasing mean fitness. Here, we discuss four key findings and how they might relate to specific characteristics of our focal species, *S. faberi*.

### Addition of N did not strengthen selection favoring trait values associated with greater light acquisition

We found no evidence that N addition strengthened selection favoring trait values associated with greater light acquisition in *S. faberi*, as selection for height and leaf count did not differ between treatments, and selection favoring lower SLA (thicker leaves) was stronger in the addition treatment. Moreover, increased light asymmetry was associated with more intense selection favoring lower SLA (rather than higher as predicted). These results are surprising given that species with greater light acquisition capabilities are often favored under N addition at the community level (Tatarko and Knops, 2018). Several factors might explain why N addition did not increasingly favor trait values associated with greater light acquisition. Other environmental variables besides light may have been important selective agents. Lower species diversity was associated with stronger selection favoring greater height and stronger selection favoring lower SLA (Figure 3B, F), suggesting this aspect of the environment may be more important for shaping patterns of selection on these traits. Other unmeasured variables may also have been important for shaping selection. For example, water availability is a key factor influencing plant evolution, and potential water savings from thicker leaves may be more important for fitness than increased light acquisition (Poorter et al., 2009). Additionally, observed patterns of selection may be in part due to environmental covariances between traits and fitness (Rausher, 1992). For example, unfavorable, low‐light conditions may plastically increase SLA while also reducing fitness, creating an apparent causal trait–fitness relationship. The negative relationship between light asymmetry and SLA selection differentials (Figure 3E) is consistent with a shade‐mediated covariance between SLA and fitness. Although such environmental covariances rarely appear to have strong effects on estimates of selection (Stinchcombe et al., 2002), our next step is to plant pedigreed populations into this N addition LTER experiment to conduct genotypic selection analyses that reduce the influence of such environmental covariances on estimates of selection.

### Effects of N addition on selection are inconsistent with trait shifts in response to N

Nitrogen enrichment alters the distribution of plant traits in the community through both changes in species abundances (i.e., species sorting; e.g., Suding et al., 2005) and shifts in species trait values (i.e., intraspecific trait value shifts; Lepš et al., 2011; La Pierre and Smith, 2015; Siefert and Ritchie, 2016; Tatarko and Knops, 2018). As described above, one might expect that the same traits favored at the community scale are also favored within populations, yet our estimates of natural selection are inconsistent with the commonly observed phenomenon that species with traits that enhance light acquisition are favored by N addition. Instead, perhaps intraspecific trait value shifts better predict patterns of natural selection as these trait shifts may be due to evolutionary responses to long‐term N addition treatments as well as more rapid plastic responses to the environment. Indeed, it has been observed that community‐level trait value shifts due to species sorting can oppose intraspecific trait value shifts in response to elevated N (Kichenin et al., 2013; Siefert and Ritchie, 2016), and evolutionary responses to even small‐scale nutrient additions have been observed in past long‐term experiments (Snaydon and Davies, 1982; Silvertown et al., 2009).

In this study, *S. faberi*, a species that has increased its abundance an average of 144% in response to 30 years of N addition (i.e., an “enriched” species; La Pierre and Smith, 2015), exhibited greater height, leaf count, and SLA, and delayed flowering in response to N addition. For all four traits, our measures of natural selection are inconsistent with the observed trait shifts, suggesting that phenotypic plasticity, rather than adaptive evolution, was the dominant process driving observed intraspecific trait value shifts. For example, greater mean height in the addition treatment is unlikely to be due to evolved differences because greater height was favored consistently in both addition and ambient treatments. For SLA, on which selection differed between treatments, N addition strengthened selection for low SLA, counter to the shift in mean SLA. Many previous studies have documented intraspecific trait value shifts under N addition that are consistent with mean trait value shifts observed in this study (toward greater height, leaf count, and SLA, and delayed flowering; Cleland et al., 2006; Lepš et al., 2011; Smith et al., 2012; Xia and Wan, 2013; La Pierre and Smith, 2015; Siefert and Ritchie, 2016; Yin et al., 2017; Huang et al., 2018; Tatarko and Knops, 2018; for a meta‐analysis of flowering time, see Wang and Tang, 2019). Thus, if our results are indicative of a wider pattern, then intraspecific trait value shifts under N may rarely be adaptive.

### Addition of N did not influence the opportunity for selection

We hypothesized that N addition would alter the opportunity for selection by increasing mean absolute fitness and changing the variance in absolute fitness, the latter due to stronger light asymmetry that can exacerbate differences in growth rates between competitors (Weiner, 1990; Schwinning and Weiner, 1998) or more intense herbivory on larger plants (Waterton and Cleland, 2021). Nitrogen addition did not significantly influence the opportunity for selection, despite more than tripling mean absolute fitness, which indicates that increases in absolute fitness were approximately proportional (i.e., fitness increased by a similar percentage for all individuals regardless of their relative fitness). Furthermore, we found that light asymmetry per se was not significantly associated with the opportunity for selection (Figure 5A). Instead, the opportunity for selection varied greatly within N treatments and this variation was largely explained by plots, indicating that other factors, such as species diversity (see discussion below), were the major determinants in shaping this population‐level parameter.

Our results therefore contradict the theoretical prediction that higher resource availability should decrease the opportunity for selection (Rundle and Vamosi, 1996), which is based on the assumption that gains in absolute fitness are distributed evenly among individuals in absolute, rather than proportional, terms (e.g., all individuals produce 10 more offspring). Although previous work has shown that N addition can reduce the opportunity for selection in plant populations (e.g., Mattila and Kuitunen, 2000), several other studies, like ours, have also found no change (Case and Ashman, 2007; Sletvold et al., 2017). Therefore, it appears that greater light asymmetry, a consistent result of N enrichment, does not sufficiently increase growth asymmetry among conspecifics to outweigh the effects of increases in mean fitness on the opportunity for selection. One potential reason why increased light asymmetry might not increase growth asymmetry among conspecifics, despite promoting competitive exclusion at the species level, could be that negative allometric scaling confers relative growth and metabolic advantages to smaller conspecifics that mitigate effects of reduced light availability (Enquist et al., 2007).

### Species diversity was associated with patterns of selection and the opportunity for selection

Populations of *S. faberi* in more species‐diverse communities were characterized by a lower opportunity for selection (Figure 5B) and weaker total selection favoring greater height and lower SLA (Figure 3B, F). Species diversity may affect the opportunity for selection in two, nonmutually exclusive ways: firstly, greater species diversity itself might reduce fitness disparities among conspecifics (i.e., causal effects). For example, more diverse communities may be characterized by increased resource partitioning (Mueller et al., 2013; Williams et al., 2017), with individuals competing less strongly or less often with conspecifics for which niche overlap is greater (Chesson, 2000; Adler et al., 2018). Furthermore, there is growing evidence that plant community diversity itself can select for greater niche partitioning and resource‐use complementarity, although the underlying mechanisms are not well characterized (Zuppinger‐Dingley et al., 2014; Schob et al., 2018; van Moorsel et al., 2018a, 2018b). Although inconsistent with our results, an alternative causal effect could be that greater diversity increases fitness variance because other species vary in the strength of competitive effects that they exert (i.e., “species fitness”; Chesson, 2000). Secondly, external processes that promote species coexistence in communities might also reduce fitness disparities among conspecifics (i.e., parallel effects). For example, a greater number of limiting resources can provide trade‐off axes by which species coexist (Harpole and Tilman, 2007) and might also decrease the variance in performance among conspecifics if such limiting resource trade‐off axes are similarly present within species.

Species diversity could reduce the strength of selection by making selection less consistent across individuals within the population. For example, in less‐diverse communities, individuals may face only one or a few competing species and competition with those species may select strongly for particular trait values. However, in a more diverse community, individuals within a population will be competing with many different species which may each favor different trait values. This more diffuse selection may result in weaker selection on any one particular trait for the population. Although surprisingly few studies have investigated how competitor identity affects natural selection, examples indicate that the identity of heterospecific competitors or intra‐ vs. interspecific competition can affect the strength, and sometimes even the direction, of selection on a focal plant population (Lankau and Strauss, 2008; Lau et al., 2010).

### Will N affect evolutionary processes similarly across species?

The effects of N addition on evolutionary processes that we observed in this study may be in part due to characteristics of our focal species, *S. faberi*, a highly selfing, annual grass that strongly benefits from N addition. For example, one might expect stronger effects on outcrossing taxa that harbor greater genetic (and likely phenotypic) variation. While among‐population genetic variation has been documented in *S. faberi* for phenological, morphological, and growth traits, each population harbored low genetic diversity likely due to the high selfing rate of this species (>97%; Warwick et al., 1987; Volenberg and Stoltenberg, 2002). Genetic diversity might be similarly low in our plots and such low within‐plot genetic diversity may reduce phenotypic variance and thus limit our ability to detect effects of N addition on patterns of selection. Observed results for phenotypic selection and the opportunity for selection in *S. faberi* also might differ from those in “baseline” species that decrease in abundance in response to N addition (La Pierre and Smith, 2015). For example, given that stronger light asymmetry drives the loss of species from communities (Hautier et al., 2009; DeMalach et al. 2016, 2017b, 2017a; Zhang et al., 2020; Xiao et al., 2021), selection favoring trait values associated with light acquisition (e.g., increased height and SLA) may be most intense in such baseline species (Reiss, 2012). We suggest that characterizing how N‐mediated effects on selection and the opportunity for selection differ between enriched and baseline species could provide further insight into the underlying mechanisms contributing to evolutionary responses to N addition.

## CONCLUSIONS

Our results illustrate that N enrichment can be a potent agent of selection, just as it is a strong determinant of plant community composition, diversity, and function. However, the effects of N on patterns of selection and the opportunity for selection may be difficult to predict, whether based on theory (e.g., predicting effects of N on the opportunity for selection based on mean fitness) or previous empirical work (e.g., predicting effects of N on selection based on changes in light asymmetry and the traits favored at the community level). With future inputs of anthropogenic N expected to continue rising (Lamarque et al., 2005), characterizing the evolutionary consequences of this chronic disturbance will be increasingly necessary to understanding its long‐term impacts on communities and ecosystems.

## AUTHOR CONTRIBUTIONS

J.W. and J.L. conceived the ideas and designed the methodology, J.W. and M.H. collected the data, and J.W. led the writing of the manuscript.

## Supporting information


**Appendix S1**. The opportunity for selection shown as the relationship between squared mean absolute fitness and the variance in absolute fitness and hypothetical scenarios in which increased mean fitness reduces, has no effect, and increases the opportunity for selection.

**Appendix S2**. Supplemental methods.

**Appendix S3**. Scatterplot of the relationship between total main panicle fruit count and total main panicle length.

**Appendix S4**. Histogram of fruit counts.

**Appendix S5**. Estimates of total and direct phenotypic nonlinear selection coefficients and their differences in ambient and addition N treatments.

**Appendix S6**. Mean values of traits and fitness components in ambient and addition N treatments.

**Appendix S7**. Phenotypic correlations and their statistical significance for individuals in each subplot in ambient and addition N treatments.

## Data Availability

Data, metadata, and the R script for reproducing data analyses and figures are deposited in Dryad Digital Repository: https://doi.org/10.5061/dryad.59zw3r2bg (Waterton et al., 2022).
