# Supplementary material for: Evolutionary effects of nitrogen are not easily predicted from ecological responses
Source: Am J Bot. 2022 Nov 13;109(11):1741–56. doi: 10.1002/ajb2.16095 (PMC10099611; doi:10.1002/ajb2.16095)
Supplement: Supplementary file 1 — Appendix S1. The opportunity for selection shown as the relationship between squared mean absolute fitness and the variance in absolute fitness and hypothetical scenarios in which increased mean fitness reduces, has no effect, and increases the opportunity for selection. [file AJB2-109-1741-s004.docx]

**Appendix S1.** The opportunity for selection shown as the relationship between squared mean absolute fitness ($\bar{W}^{2}$) and the variance in absolute fitness ($\sigma_{W}^{2}$) and hypothetical scenarios in which increased mean fitness (A) reduces, (B) has no effect, and (C) increases the opportunity for selection. The opportunity for selection equals the ratio $\frac{\sigma_{W}^{2}}{\bar{W}^{2}}$, and the grey dashed line is the identity line at which $\frac{\sigma_{W}^{2}}{\bar{W}^{2}}$ = 1. The grey circle represents a population before an increase in mean fitness (e.g., due to nutrient enrichment); black circles represent the population after the increase in mean fitness under each scenario. (A) Fitness gains are equal in absolute terms (e.g., each individual produces 10 more offspring) such that $\sigma_{W}^{2}$ is unchanged. (B) Fitness gains are proportional (e.g., each individual increases their fitness by 10%) such that $\sigma_{W}^{2}$ increases proportionally to $\bar{W}^{2}$. Note that the opportunity for selection is constant as long as the ratio $\frac{\sigma_{W}^{2}}{\bar{W}^{2}}$ remains unchanged (i.e., the vector is parallel to the identity line). (C) Fitness gains are disproportionally higher for the most fit individuals such that the increase in $\sigma_{W}^{2}$ outweighs the increase in $\bar{W}^{2}$ and so $\frac{\sigma_{W}^{2}}{\bar{W}^{2}}$ > 1.

**
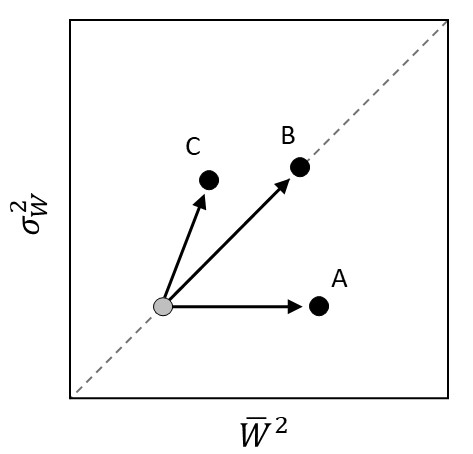
**
