# Supplementary material for: Evolutionary effects of nitrogen are not easily predicted from ecological responses
Source: Am J Bot. 2022 Nov 13;109(11):1741–56. doi: 10.1002/ajb2.16095 (PMC10099611; doi:10.1002/ajb2.16095)
Supplement: Supplementary file 2 — Appendix S2. Supplemental methods. [file AJB2-109-1741-s001.docx]

**Appendix S2. Supplemental methods.**

***Specific leaf area (SLA) measurements***

To check for potential effects of leaf aging or senescence on SLA, before drying, we gave each leaf a color score between 1 (leaf without any green) and 4 (fully green leaf). The linear regression of SLA against leaf color scores was significant and positive (*β* = 1.01, *t* = 3.65, *P* < 0.001), opposite what would be expected if the onset of leaf senescence resulted in significant mass loss. Regardless, the adjusted *R*^2^ of 0.015 suggests that leaf condition had minimal influence over SLA.

***Community metrics***

To quantify aboveground productivity and species diversity in microplots, we harvested annual net primary productivity (ANPP) in each microplot between 22–29 September (approximately at peak biomass). Within a 0.5 × 2 m area in the central 3 × 3 m of each microplot, we clipped all live plants at ground surface and sorted them according to species. The full protocol for the annual ANPP harvest in T7 plots can be found at <https://www.lter.kbs.msu.edu/protocols/143>. For each microplot, we calculated total ANPP (g m^−2^) as well as the Shannon–Wiener diversity index ($H^{'}$) according to the following equation:

$H^{'}= -\sum_{i=1}^{s} p_{i}\ln p_{i},$

where $p_{i}$ is the proportion of total ANPP represented by species $i$, and $s$ is the number of species in the sample. When calculating $H^{'}$, we excluded unsorted biomass that could not be identified as belonging to unique species (this constituted between 3–14% of total ANPP in each microplot). We calculated $H^{'}$ with the diversity function in the package vegan (Oksanen et al., 2020).

To quantify light asymmetry in microplots, we measured photosynthetically active radiation (PAR) using an AccuPAR LP80 PAR Ceptometer (Decagon Devices, Pullman, WA, USA) at five heights: ground level (0% canopy height), 25%, 50%, and 75% canopy height, and at the canopy top (100% canopy height). We took two measurements at each height—one with the ceptometer facing East–West and the other North–South—in the southwest quadrant of each microplot. We took these measurements within an hour of the solar zenith on clear days between 30 August and 4 September. For each microplot, we extracted the slope of a linear regression of log PAR (μmol m^−2^ s^−1^) against height (cm) and used this as a measure of light asymmetry (DeMalach et al., 2017).

***Statistical analyses***

*Nonlinear selection*

We estimated standardized selection differentials (quadratic: *C_ii_*) that describe total selection in each N treatment as the regression coefficients from separate linear mixed models (LMMs) for each trait of within-subplot relative fecundity predicted by linear/quadratic trait terms, with random slopes for linear/quadratic trait terms within subplots. We estimated standardized selection gradients (quadratic: γ*_ii_*; cross-product: γ*_ij_*) that describe direct selection in each N treatment as the partial regression coefficients from LMMs of within-subplot relative fecundity predicted by linear/quadratic/cross-product trait terms, with random slopes for each linear/quadratic trait term within subplots. We doubled quadratic regression coefficients to obtain quadratic selection differentials and gradients (Stinchcombe et al., 2008). Due to violations of LMM assumptions that would otherwise render significance tests unreliable (Mitchell-Olds and Shaw, 1987; Schielzeth et al., 2020), we obtained 95% confidence intervals for selection coefficients and the difference between selection coefficients in each N treatment with nonparametric bootstrapping. For each N treatment, we used the nonparametric cases bootstrap to resample individuals within each subplot to generate 1000 bootstrap samples that maintain the clustered structure of the data using the cases_bootstrap function in the package lmeresampler (Loy et al., 2022). We used the percentile method to estimate 95% confidence intervals for selection coefficients as bootstrap distributions were approximately symmetric about their means (Efron and Tibshirani, 1993), with a selection coefficient deemed significant if the 95% confidence interval did not contain 0. To obtain 95% confidence intervals for the difference in selection coefficients between N treatments, we calculated the difference between the selection coefficients from each of the 1000 independent bootstrap samples in each N treatment. The percentile method was used to calculate the 95% confidence interval for the difference in selection coefficients, which were deemed to differ significantly between N treatments if the 95% confidence interval did not contain 0.

*Associations between community metrics and linear selection*

To test whether total and direct linear selection in each subplot was associated with each community metric (light asymmetry, species diversity, and herbivory intensity), we first extracted standardized selection differentials (*S_i_*) and gradients (*β_i_*) for each subplot from separate ordinary least squares/multiple regressions of within-subplot relative fecundity against within-subplot standardized traits (single trait models for differentials, all four trait models for gradients) using the base lm function. We used the four selection differentials and gradients from each subplot as the response variable in six separate PERMANOVAs (one for each community metric for each of differentials and gradients) fit with the adonis2 function in the package vegan (Oksanen et al., 2020). In each PERMANOVA, the Euclidean distance among selection gradients among subplots was predicted by either light asymmetry, species diversity, or herbivory intensity, with plot as a blocking factor. In the case that PERMANOVA revealed significant effects of a community metric on selection (see Results), we then fit separate univariate LMMs with selection differentials/gradients for each trait in subplots predicted by the relevant community metric and with random intercepts for plot.

*N effects on phenotypic correlations*

To describe the multivariate phenotypes available to selection, we estimated phenotypic correlations in each N treatment while including incomplete observations (i.e., any individual with values for two or more traits). To formally test how N addition influenced phenotypic correlations, we estimated correlations separately for each subplot and used these as the response variable in a PERMANOVA. In the PERMANOVA, the Euclidean distance among trait correlations among subplots was predicted by N treatment with plot as a blocking factor. This method is similar to the method for comparing genetic variance–covariance matrices (**G**) devised by Roff (2002), but using nonparametric PERMANOVA instead of MANOVA and using separate estimates from each subplot instead of jackknife pseudovalues from a single group. In the case that PERMANOVA revealed significant effects of N treatment (see Results), we then fit separate univariate LMMs with phenotypic correlations in subplots predicted by N and with random intercepts for plot.

**References**

DeMalach, N., E. Zaady, and R. Kadmon. 2017. Light asymmetry explains the effect of nutrient enrichment on grassland diversity. *Ecology Letters* 20: 60–69.

Efron, B., and R. J. Tibshirani. 1993. An introduction to the bootstrap. Chapman & Hall, NY, NY, USA.

Loy, A., S. Steele, and J. Korobova. 2022. lmeresampler: bootstrap methods for nested linear mixed-effects models [R package version 0.2.2]. Website <https://cran.r-project.org/web/packages/lmeresampler>.

Mitchell-Olds, T., and R. G. Shaw. 1987. Regression analysis of natural selection: statistical inference and biological interpretation. *Evolution* 41: 1149-1161.

Oksanen, J., F. G. Blanchet, M. Friendly, R. Kindt, P. Legendre, D. McGlinn, P. R. Minchin, et al. 2020. vegan: community ecology package [R package version 2.5-7]. Website <https://CRAN.R-project.org/package=vegan>.

Roff, D. 2002. Comparing **G** matrices: a MANOVA approach. *Evolution* 56: 1286-1291.

Schielzeth, H., N. J. Dingemanse, S. Nakagawa, D. F. Westneat, H. Allegue, C. Teplitsky, D. Réale, et al. 2020. Robustness of linear mixed‐effects models to violations of distributional assumptions. *Methods in Ecology and Evolution* 11: 1141-1152.

Stinchcombe, J. R., A. F. Agrawal, P. A. Hohenlohe, S. J. Arnold, and M. W. Blows. 2008. Estimating nonlinear selection gradients using quadratic regression coefficients: double or nothing? *Evolution* 62: 2435–2440.
