# Supplementary material for: Evolutionary effects of nitrogen are not easily predicted from ecological responses
Source: Am J Bot. 2022 Nov 13;109(11):1741–56. doi: 10.1002/ajb2.16095 (PMC10099611; doi:10.1002/ajb2.16095)
Supplement: Supplementary file 3 — Appendix S3. Scatterplot of the relationship between total main panicle fruit count and total main panicle length. [file AJB2-109-1741-s007.docx]

**Appendix S3. Scatterplot of the relationship between total main panicle fruit count and total main panicle length.**

**
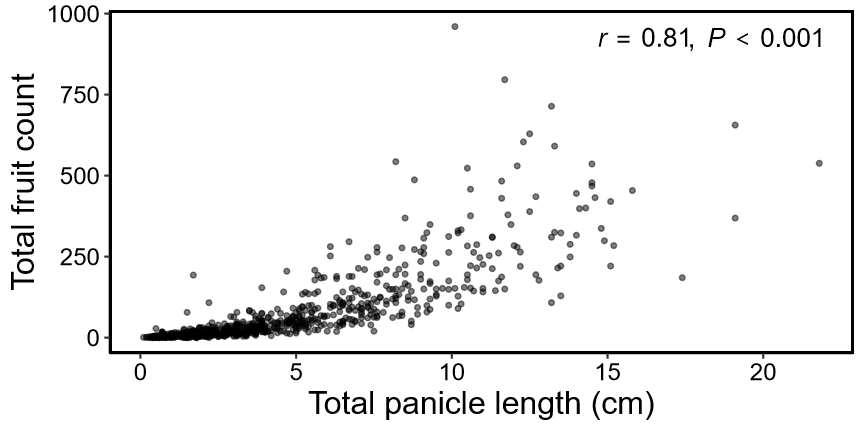
**
