# Supplementary material for: Evolutionary effects of nitrogen are not easily predicted from ecological responses
Source: Am J Bot. 2022 Nov 13;109(11):1741–56. doi: 10.1002/ajb2.16095 (PMC10099611; doi:10.1002/ajb2.16095)
Supplement: Supplementary file 4 — Appendix S4. Histogram of fruit counts. [file AJB2-109-1741-s005.docx]

**Appendix S4. Histogram of fruit counts.** Fruit counts of 0 are indicated by the black line; all other bins have width = 5.

**
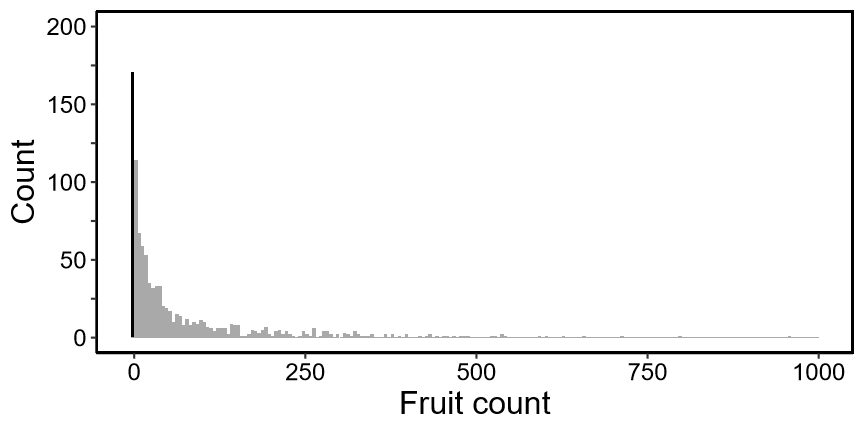
**
