# Supplementary material for: Evolutionary effects of nitrogen are not easily predicted from ecological responses
Source: Am J Bot. 2022 Nov 13;109(11):1741–56. doi: 10.1002/ajb2.16095 (PMC10099611; doi:10.1002/ajb2.16095)
Supplement: Supplementary file 5 — Appendix S5. Estimates of total and direct phenotypic nonlinear selection coefficients and their differences in ambient and addition N treatments. [file AJB2-109-1741-s003.docx]

**Appendix S5. Estimates of total and direct phenotypic nonlinear selection coefficients and their differences in ambient and addition N treatments.** Values are coefficients from models predicting within-subplot relative fecundity (i.e., selection differentials [quadratic: *C_ii_*] for total selection and selection gradients [quadratic: *γ_ii_*; cross-product: *γ_ij_*] for direct selection). We obtained 95% confidence intervals for selection coefficients and differences between N treatments with nonparametric bootstrapping. Values in bold indicate significant selection or selection that differed significantly between N treatments (i.e., 95% confidence interval does not contain 0).

| **Trait** | **N treatment** | | **N-mediated difference in selection coefficient (Addition** − **Ambient)** |
| --- | --- | --- | --- |
|  | **Ambient** | **Addition** |  |
| Total selection | | | |
| Height (*C_ii_*) | **0.99**  (0.76, 1.23) | **0.80**  (0.63, 0.99) | −0.19  (−0.49, 0.12) |
| Leaf count (*C_ii_*) | **0.23**  (0.031, 0.49) | −0.056  (−0.25, 0.10) | **−0.28**  (−0.63, −0.028) |
| SLA (*C_ii_*) | **0.14**  (0.003, 0.51) | **0.75**  (0.59, 1.03) | **0.61**  (0.24, 0.92) |
| Flowering date (*C_ii_*) | **0.37**  (0.22, 0.56) | **0.24**  (0.050, 0.48) | −0.14  (−0.40, 0.15) |
| Direct selection | | | |
| Height (*γ_ii_*) | **0.85**  (0.27, 1.58) | 0.27  (−0.52, 0.80) | −0.57  (−1.73, 0.18) |
| Leaf count (*γ_ii_*) | 0.23  (−0.37, 0.63) | 0.065  (−0.27, 0.27) | −0.17  (−0.71, 0.44) |
| SLA (*γ_ii_*) | 0.033  (−0.050, 0.15) | −0.085  (−0.40, 0.29) | −0.12  (−0.42, 0.27) |
| Flowering date (*γ_ii_*) | −0.071  (−0.26, 0.24) | −0.044  (−0.28, 0.16) | 0.027  (−0.35, 0.30) |
| Height × leaf count (*γ_ij_*) | 0.14  (−0.36, 0.55) | 0.20  (−0.037, 0.50) | 0.069  (−0.38, 0.63) |
| Height × SLA (*γ_ij_*) | −0.12  (−0.37, 0.16) | −0.52  (−1.25, 0.095) | −0.40  (−1.22, 0.21) |
| Height × flowering date (*γ_ij_*) | 0.036  (−0.18, 0.44) | 0.12  (−0.19, 0.38) | 0.087  (−0.45, 0.38) |
| Leaf count × SLA (*γ_ij_*) | 0.25  (−0.19, 0.49) | 0.078  (−0.22, 0.43) | −0.18  (−0.48, 0.43) |
| Leaf count × flowering date (*γ_ij_*) | 0.15  (−0.24, 0.40) | **0.25**  (0.073, 0.41) | 0.093  (−0.21, 0.52) |
| SLA × flowering date (*γ_ij_*) | 0.033  (−0.20, 0.21) | 0.067  (−0.19, 0.33) | 0.034  (−0.26, 0.39) |
