# Supplementary material for: Evolutionary effects of nitrogen are not easily predicted from ecological responses
Source: Am J Bot. 2022 Nov 13;109(11):1741–56. doi: 10.1002/ajb2.16095 (PMC10099611; doi:10.1002/ajb2.16095)

**Appendix S6. Mean values of (A–D) traits and (E, F) fitness components in ambient and addition N treatments.** *P* values are from linear mixed models or generalized linear mixed models of traits or fitness components predicted by N treatment. Solid horizontal lines show the estimated marginal means across subplots. Filled circles show subplot mean values and dotted lines connect subplots in the same plot.


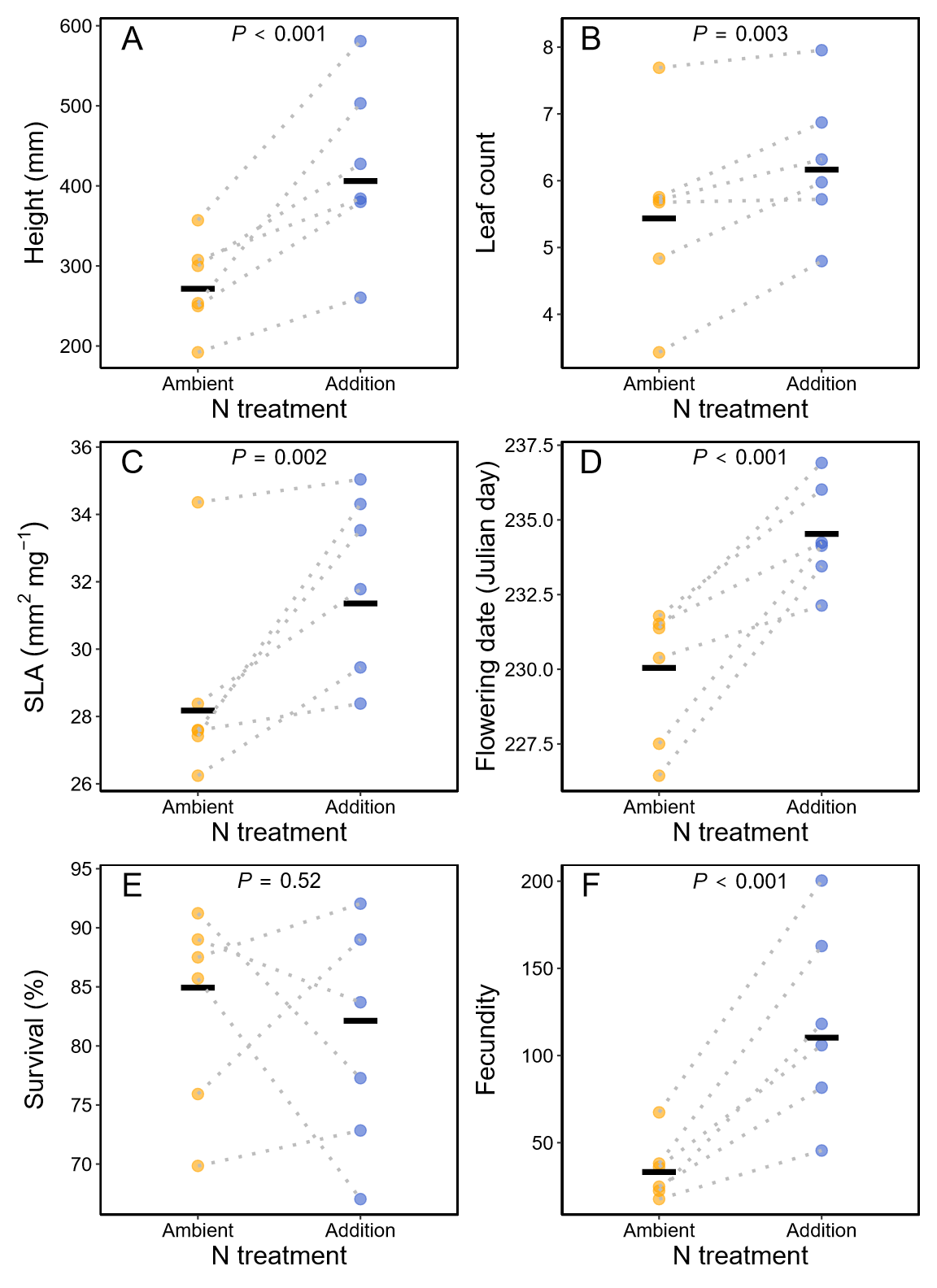

Supplement: Supplementary file 6 — Appendix S6. Mean values of traits and fitness components in ambient and addition N treatments. [file AJB2-109-1741-s002.docx]
