# Supplementary material for: Evolutionary effects of nitrogen are not easily predicted from ecological responses
Source: Am J Bot. 2022 Nov 13;109(11):1741–56. doi: 10.1002/ajb2.16095 (PMC10099611; doi:10.1002/ajb2.16095)
Supplement: Supplementary file 7 — Appendix S7. Phenotypic correlations and their statistical significance for individuals in each subplot in ambient and addition N treatments. [file AJB2-109-1741-s006.docx]

**Appendix S7. Phenotypic correlations and their statistical significance for individuals in each subplot in ambient (above diagonal) and addition (below diagonal) N treatments.** Significance: †*P* < 0.1, **P* < 0.05, ***P* < 0.01, ****P* < 0.001.

| **Plot 1** | | | | |
| --- | --- | --- | --- | --- |
|  | Height | Leaf count | SLA | Flowering date |
| Height |  | 0.77*** | −0.18 | −0.51*** |
| Leaf count | 0.81*** |  | −0.22 | −0.54*** |
| SLA | −0.55*** | −0.40** |  | 0.18 |
| Flowering date | −0.66*** | −0.54*** | 0.71*** |  |
| **Plot 2** | | | | |
|  | Height | Leaf count | SLA | Flowering date |
| Height |  | 0.79*** | −0.69*** | −0.76*** |
| Leaf count | 0.67*** |  | −0.62*** | −0.66*** |
| SLA | −0.87*** | −0.60*** |  | 0.59*** |
| Flowering date | −0.71*** | −0.58*** | 0.68*** |  |
| **Plot 3** | | | | |
|  | Height | Leaf count | SLA | Flowering date |
| Height |  | 0.85*** | 0.013 | −0.79*** |
| Leaf count | 0.71*** |  | −0.059 | −0.64*** |
| SLA | −0.88*** | −0.60*** |  | −0.019 |
| Flowering date | −0.73*** | −0.51*** | 0.77*** |  |
| **Plot 4** | | | | |
|  | Height | Leaf count | SLA | Flowering date |
| Height |  | 0.72*** | −0.58*** | −0.80*** |
| Leaf count | 0.69*** |  | −0.48*** | −0.63*** |
| SLA | −0.73*** | −0.48*** |  | 0.48*** |
| Flowering date | −0.85*** | −0.57*** | 0.75*** |  |
| **Plot 5** | | | | |
|  | Height | Leaf count | SLA | Flowering date |
| Height |  | 0.84*** | −0.61*** | −0.57*** |
| Leaf count | 0.81*** |  | −0.44*** | −0.48*** |
| SLA | −0.73*** | −0.60*** |  | 0.43*** |
| Flowering date | −0.72*** | −0.69*** | 0.51*** |  |
| **Plot 6** | | | | |
|  | Height | Leaf count | SLA | Flowering date |
| Height |  | 0.67*** | 0.072 | −0.74*** |
| Leaf count | 0.67*** |  | −0.009 | −0.61*** |
| SLA | −0.77*** | −0.53*** |  | −0.061 |
| Flowering date | −0.73*** | −0.53*** | 0.56*** |  |
